# A Scoping Review on Gaps in the Diagnostic Criteria for Proliferative Verrucous Leukoplakia: A Conceptual Proposal and Diagnostic Evidence-Based Criteria

**DOI:** 10.3390/cancers13153669

**Published:** 2021-07-21

**Authors:** Miguel Ángel González-Moles, Pablo Ramos-García, Saman Warnakulasuriya

**Affiliations:** 1 School of Dentistry, University of Granada, 18010 Granada, Spain; 2 Instituto de Investigación Biosanitaria ibs.GRANADA, 18012 Granada, Spain; 3 WHO Collaborating Group for Oral Cancer, 1211 Geneva, Switzerland; 4 Faculty of Dentistry, Oral and Craniofacial Sciences, King’s College London, London SE1 9RT, UK; saman.warne@kcl.ac.uk

**Keywords:** proliferative verrucous leukoplakia, oral cancer, early diagnosis, diagnostic criteria, scoping review

## Abstract

**Simple Summary:**

Proliferative verrucous leukoplakia is considered as an oral potentially malignant disorder that presents the highest tendency to recurrence and malignant transformation, alt-hough published diagnostic criteria are inconsistent. A precise evidence-based diagnosis is im-portant to differentiate this lesion from others on the oral mucosa with less tendency for cancer progression, and thus establish specific management protocols aimed at the early diagnosis of oral cancer. In this scoping review the published conceptual and diagnostic criteria for prolifera-tive verrucous leukoplakia were comprehensively analyzed, and a conceptual proposal for fu-ture diagnosis is proposed based on current evidence.

**Abstract:**

Proliferative verrucous leukoplakia (PVL) is considered as an oral potentially malignant disorder (OPMD) that presents with a high tendency to recurrence after treatment and has the highest malignant transformation ratio among all OPMD (50%). Evidence-based publications have indicated that the malignant evolution reported is significantly related to the inconsistent diagnostic criteria used in primary-level studies; so, it has been hypothesized that the risk of oral cancer for this disease could even be underestimated. This is important because PVL requires specific management protocols, evidence-based, aimed at the early diagnosis of cancer developing in these lesions. We present a scoping review—a novel approach to mapping the available literature on a given topic to provide an overview of the available research evidence and to highlight possible gaps in the evidence—especially related in our study to the diagnostic aspects of PVL, and to issue a conceptual proposal and diagnostic criteria for PVL. We conclude that PVL is a white, multifocal and progressive lesion with a high malignant transformation rate which is diagnosed mainly around the age of 60 years without any specific histological characterization. We also advise a personal reflection on the level of certainty with which the clinician makes the diagnosis of a particular case of PVL.

## 1. Introduction

The recent consensus meeting of the WHO Collaborating Center for Oral Cancer held in Glasgow, Scotland in 2020 [1], focused on updating concepts and classification of oral potentially malignant disorders (OPMD). Proliferative verrucous leukoplakia (PVL) was classified as one of the important entities included among the wide range of conditions considered as OPMDs. The Working Group defined PVL as a distinct form of multifocal oral leukoplakia characterized by having a progressive clinical course, changing clinical, and histopathological features and is associated with the highest proportion of oral cavity cancer development compared with other OPMD. The essential characteristics of this OPMD are its recognized nature of recurrence on complete removal and high frequency of development of oral carcinoma in comparison with other OPMDs [2]. Thus, in a recent systematic review published in 2020, a malignancy transformation rate of 49.5% (95% CI = 26.7–72.4%) has been reported [3]. An important consequence of this OPMD is its marked tendency to evolve into multiple carcinomas, due to underlying field cancerization of the affected mucosa [4]. A subgroup of participants and collaborators at the aforementioned consensus meeting were commissioned to update the information regarding the malignant transformation rate of PVL and factors affecting it, which was carried out through a systematic review and meta-analysis [5]. The results of this study yielded a malignancy transformation rate of 43.87% (95% CI = 31.93–56.13). A remarkable result of the Ramos-García et al. paper is related to the high variability of the malignancy rates found in the studies included in this meta-analysis, which ranged between 0% [6] and 100% [7], and excluding these extreme values, between 14% [8] and 75% [9]. The analysis of the reasons for this variability allowed us to point out the low methodological quality of some of the 17 studies meta-analyzed in this paper [5]. Further analyses, after applying the relevant sensitivity analyses, revealed that those studies with lower methodological quality tended to report lower PVL malignant transformation rates (27.60%, 95% CI = 12.86–44.68). Among the factors limiting the methodological quality of papers meta-analyzed by Ramos-García et al. [5] were the criteria used by respective authors for the PVL diagnosis in their original research and the short follow-up times reported. To date, four research groups have proposed diagnostic criteria for PVL [10,11,12,13] (Table 1). In our opinion, the diagnostic criteria used could influence the reported rate of PVL malignancy and most of the proposed criteria could allow inclusion of lesions that are not really PVL [5] resulting in lower malignant transformation rates. We believe that the absence of evidence-based diagnostic criteria is at the origin of the variability of the PVL malignant transformation rates reported in the literature.

In this study we present a scoping review—a novel approach for mapping available literature on a given topic to provide an overview of the available research evidence and to highlight potential gaps in the evidence [14,15]. Here we critically review the clinical and histological aspects considered as the diagnostic criteria for PVL recommended in the four proposals that have been published to date. We drew up a list of research questions derived from the published diagnostic criteria. The aim of the study was to evaluate the extent to which the authors adhered to the diagnostic criteria given in the original proposals and whether low, intermediate, and high MT rates reflect adherence to the diagnostic criteria selected by the authors. Our analysis allowed us to propose a concept for the future diagnosis of PVL in which a minimum of evidence-based diagnostic criteria is implicit that should be met to consider a white lesion as a PVL.

## 2. Materials and Methods

Since several non-evidence-based PVL diagnostic criteria have been published, a scoping review design seems pertinent to rigorously synthesize evidence, guide future research and make recommendations [14,15]. This scoping review closely followed the Preferred Reporting Items for Systematic reviews and Meta-Analyses extension for Scoping Reviews (PRISMA-ScR) [16].

### 2.1. Protocol

Despite the lack of international consensus on the a priori design of study protocol of a scoping review, in order to minimize risk of bias and improve the transparency, precision, and integrity of our scoping review, a protocol on its methodology was designed (design date: February 2021). A copy of the study protocol can be found as supplementary material (File S1).

### 2.2. Search Strategy

PubMed, Embase, Web of Science, and Scopus databases were searched for studies published before February 2021, with no lower date limit. In order to maximize sensitivity, this search was conducted by combining the keywords “proliferative” and “verrucous” and “leukoplakia”. Thesaurus terms (e.g., MeSH or Emtree) were not used due to the lack of specific terms for the target disease (i.e., proliferative verrucous leukoplakia). We also manually screened the reference lists of retrieved studies looking for additional relevant studies and consulted experts in the field. All references were managed using Mendeley v. 1.19.4 (Elsevier. Amsterdam, The Netherlands); duplicate references were eliminated.

### 2.3. Eligibility Criteria

Following the Condition, Context and Population CoCoPop framework—designed by Joanna Briggs Institute, University of Adelaide, Australia (Aromataris and Munn, 2020)—the following inclusion criteria were applied: studies investigating the malignant transformation potential (condition) of subjects with PVL (population) diagnosed by clinical and/or histopathological criteria (context), and their related characteristics (e.g., sex, age, tobacco use, anatomical sites affected and age of the lesions, clinical course, resistance to treatment, etc.), assessed through cohorts with follow up, without restrictions by publication language or date.

The following exclusion criteria were applied: (1) studies not investigating PVL, researching other types of OPMD or not reporting separated data on PVL; (2) studies not researching the malignant transformation potential of PVL; (3) studies only focused on gingival PVLs; (4) interventional studies, cross-sectional, case reports, reviews or meta-analyses, personal opinions or commentaries, hypotheses, protocols, letters, posters, meeting abstracts, and preclinical research (animal experimentation and in vitro studies); (5) overlapping populations; when results were derived from the same study population, we included the most recently reported or those providing more data; the use of the same population in different studies was determined by verifying the name and affiliation of the authors, source of patients, and recruitment period.

### 2.4. Study Selection Process

Eligibility criteria were independently applied by two authors (MAGM and PRG). Articles were selected in two phases, first screening titles and abstracts for articles apparently meeting inclusion criteria, and then reading the full text of selected articles, excluding those that failed to meet the eligibility criteria. Any discrepancies were resolved by consensus. The inter-agreement between evaluators on study eligibility was calculated using Cohen’s kappa (κ) statistic [17], obtaining an almost perfect agreement (99.41% of agreement, κ = 0.96).

### 2.5. Data Extraction

Two authors (MAGM and PRG) extracted data from the selected articles, completing a data collection form in a standardized manner using Excel and Word (v. 16/2018, Microsoft. Redmond, WA, USA). Data expressed as order statistics (i.e., median, interquartile range and/or maximum–minimum values) were computed and transformed into means ± standard deviation (SD) using the methods proposed by Luo et al. (2018) and Wan et al. (2014) [18,19]. If it was desirable to combine two different datasets expressed as means ± SDs from subgroups into a single group, the formula provided by Cochrane Handbook was applied [20]. Data were gathered on the first author, publication year, country and continent, publication language, sample size, study design, recruitment and follow up period, study design, diagnostic criteria, location of PVLs per patients (patients were chosen as analysis units due to higher translational potential, not lesions), sex and age of patients, tobacco and alcohol consumption. Finally, the data required to answer our set of designed questions (see next section) were also collected.

### 2.6. Critical Analysis and Evidence Synthesis

Three authors (MAGM, PRG, SW) designed and developed descriptive questions, grouped in a matrix format and based on topic areas, to search for evidence-based results and potential evidence gaps. The question matrix was built by carefully analyzing all the aspects and topics addressed in the different published diagnostic criteria. The questions critically appraised for the diagnostic criteria contained in the 4 published proposals can be found in Table 2. As the topics were identified by us, they were classified—logical and rational way—within a question, indicating the origin of these criteria. We then examined whether the individual studies applied these diagnostic criteria during case selection for their original research. Finally, we subgrouped the selected studies based on the reported MT rates to low, intermediate and high and manually analyzed whether the authors in each MT group followed the diagnostic criteria they intended to use. The authorship team subsequently synthetized and discussed results and issues to reach consensus for each question. Evidence-based results were obtained, and potential evidence gaps were noted where insufficient research evidence exists about a particular topic to make recommendations or formulate statements.

## 3. Results

### 3.1. Results of the Literature Search

**Figure 1:**
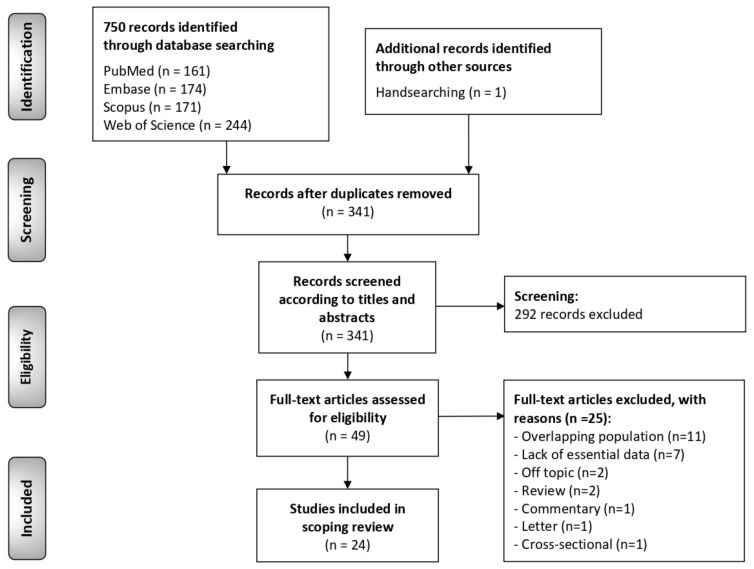
Flow diagram of the identification and selection process of the studies included in this scoping review.

The flow diagram (Figure 1) depicts the results obtained in the study identification and selection process. A total of 750 publications were retrieved: 244 from Web of Science, 174 from Embase, 171 from Scopus, 161 from PubMed and 1 handsearching the reference lists. After duplicates removal, 341 records were considered potentially eligible and screened according to titles and abstracts, leaving a sample of 49 studies for full text evaluation. Finally, 24 studies meeting all eligibility criteria were included for critical analysis and evidence synthesis in our scoping review [4,6,7,8,9,10,13,21,22,23,24,25,26,27,28,29,30,31,32,33,34,35,36,37].

### 3.2. Study Characteristics

Table 3 summarizes the characteristics of the 24 selected studies, which reported on a total of 631 patients with PVL. Sample sizes ranged between 3 and 81 patients. The studies were conducted in Europe (*n* = 12; UK [*n* = 6], Spain [*n* = 3], Italy [*n* = 2], and France [*n* = 1]), Asia (*n* = 3; India [*n* = 1], Israel [*n* = 1], and Malaysia [*n* = 1]), North America (*n* = 7; all in USA), South America (*n* = 1; from Brazil) and one multicentric international study (Brazil–USA). In relation to diagnostic criteria, 7 studies followed Hansen’s criteria, 2 studies Cerero-Lapiedra’s criteria, 2 studies Villa’s criteria, 1 study Carrard’s criteria, 8 studies used their own criteria, and 4 studies did not report their criteria. According to their study design, 23 were retrospective cohorts and only one was prospective.

### 3.3. Critical Analysis and Evidence Synthesis

The results related to the answers to the research questions posed in this scoping review are shown in Table 4. It refers to the extent to which the four proposals that published diagnostic criteria consider these research questions as important. In Table 5 we list the number of studies that published malignant transformation of PVL that had considered these research questions relevant for the diagnosis of PVL in the patients included in their series. Table 5 also offers the results on the number of individual patients included in the total series on the aspects collected in the research questions. Among the most relevant of the published results, it stands out that most studies consider that PVL should be a white (data reported by 6/24 studies [25%]; 141/141 patients [100%]), multifocal (data reported by 6/24 studies [25%]; 74/81 patients [91.36%]) and progressive (data reported by 3/24 studies [12.5%]; 36/51 patients [70.59%]), with a high rate of malignant transformation (squamous cell carcinoma: 18/24 studies [75%], 139/472 patients [29.45%]; verrucous carcinoma: 16/24 studies [66.67%], 81/384 patients [21.09%]; papillary carcinoma: 3/24 studies, 21/98 patients [21.43%]) that appears mainly around 60 years of age (23/24 studies [95.83%], 556 patients, mean of means = 63.06 years) (Table 5).

## 4. Discussion

The PVL malignancy rates reported by the two systematic reviews and meta-analyses published to date [3,5] indicate a high risk 49.5% and 43.87%, respectively, for this OPMD. Additionally, evident is a high inter-study variability of the outcomes essentially dependent on the type of criteria used for diagnosis and the short follow-up of the patients in most of the series [5]. A first analysis of our results indicates, as will be seen below, that most of the 24 papers that report rates of PVL malignancy do not fully follow the diagnostic criteria proposed in the literature [10].

Our first research question refers to *what should be the clinical course of a white lesion of the oral mucosa that would allow it to be classified as a PVL*, or otherwise, to exclude it as a candidate for this diagnosis. Hansen et al. [10] in their descriptive paper on PVL classify the lesion as persistent, while the remaining three proposals for diagnostic criteria do not. Only 25% of the papers analyzed in this scoping review (6 out of 24) [7,9,10,21,22,23] consider the persistent nature of the lesion as an essential diagnostic criterion, while the remaining 75% (18 of 24) do not refer to this criterion. In our opinion, and derived from our own clinical experience, PVL should behave as a persistent lesion, as occurring in other forms of leukoplakia, and this is probably assumed as necessary for the diagnosis by the authors who do not specifically clarify this clinical characteristic in its description of criteria. There is no report in the literature on regression of PVL lesions and thus, it seems that the scant evidence and accumulated experience advise considering PVL as persistent lesion; so, in our opinion, this characteristic should be made explicit in a conceptual proposal.

The second research question, somehow related to the previous one, *refers to the age of the lesion as decisive in order to reach the diagnosis of PVL*. Cerero-Lapiedra et al. [11] and Carrard et al. [12] point out that the lesions should be at least 5 years old, although in Cerero’s criteria this aspect is not considered as determining. In the other two criteria propositions, it is intuited that PVL is probably present for a long time before being diagnosed, although this aspect is not stated with precision; thus, Hansen et al. [10] consider PVL as a “form of leukoplakia that begins as a white plaque of hyperkeratosis that spreads over time and becomes multifocal”, while Villa et al. [13] point out that “lesions as they progress, expand in size and/or become multifocal over time”. Five out 24 (20.83%) papers included in this scoping review [13,26,27,34,35] consider the age of the lesion as a determining criterion for the PVL diagnosis, although only McParland and Warnakulasuriya (2020) [34] and Upadhyaya et al. (2018) [35] report a minimum period of evolution time—3.4 and 3 years, respectively—to be able to accept the diagnosis of PVL, while the remaining three studies do not give specific periods of time (Villa et al. 2018 [13], Borgna et al. 2017 [27], Flores et al. 2016 [26]); 20 papers (83.33%) do not consider the age of the lesion as a required criterion. We believe that probably implicit in the diagnosis of PVL is the fact that the lesion must have existed for a period before the diagnosis is given and, thus, although based on scant evidence, clinicians possibly assume this aspect as necessary in the diagnosis.

Our third research question refers to *the clinical appearance required for the diagnosis of PVL*. Studies providing information on this issue report PVL as a combination of some of the following appearances: white plaques, progressively extending along the surface of the oral mucosa, slow growing, multifocal, smooth, fissured, ulcerated, with erythematous, exophytic and/or warty appearance. The four diagnostic proposals published to date [10,11,12,13] consider PVL as white plaque lesions; 6 out of 24 studies (25%) in our scoping review [8,9,23,28,30,31], which collect information on 141 patients, report 100% of the PVL as white plaques, while the remaining papers (18 out of 24; 75%) do not give this information. In our opinion, the consideration of a PVL as a white plaque is a key fact in the diagnosis, and it should appear in a conceptual proposal or tentative diagnostic criteria; probably most authors assume that PVL essentially manifest as white plaques, although this aspect is not expressly detailed in their criteria. Regarding the multifocality of the lesions, the four PVL diagnostic proposals consider this aspect as essential, although for Cerero-Lapiedra it is a major criterion, even though not required [11]; 6 out of 24 studies [8,9,23,30,31,35] included in our scoping review (25%) provide information in this regard, reporting multifocality in 74 of 81 patients (91.36%). In our view, this is a common form of PVL presentation that should be considered in a conceptual and diagnostic criteria proposal, however, it seems logical to assume that multifocal oral mucosa affectation only develops in PVL after a long evolution time; linked to the multifocality is the progressive nature of the PVL, understanding as such the capacity of these lesions to affect progressively extensive areas of the oral mucosa. In relation to that, all of the four PVL diagnosis proposals reported consider multifocality as mandatory for the diagnosis; only 3 out of the 24 papers [8,9,30] included in our study (12.5%) provide information in this regard, reporting 36/51 patients (70.59%) presented progressive/expansive lesions extending through large areas of the oral mucosa; in our opinion, the progressive/expansive nature of PVL is an essential characteristic in the diagnosis of the disease that should therefore be included in a conceptual or diagnostic criteria proposal, although it is also evident that only after a long follow-up of the lesions, or by a meticulous clinical history, this aspect can be revealed. An important fact of the clinical expression of PVL is its verrucous appearances in some areas of the affected oral mucosa, in such a way that even this feature has been used in naming the disease—proliferative verrucous leukoplakia; of the diagnostic proposals for PVL, only Carrard et al. [12] consider warty areas as mandatory for the diagnosis; 5 of 24 papers [8,9,23,30,31] included in our scoping review (20.83%) offer this information, reporting 22 of 61 patients presenting warty areas (36.07%). For us, warty appearance acquires importance when presented and, in our view, it should be considered as a supporting diagnostic criterion. Furthermore, it is recognized that PVL could also develop erythematous areas; only Hansen et al. [10] and Villa et al. [13] consider red areas as a possible PVL clinical appearance, albeit this is not required for diagnosis; 6 of 24 papers (25%) [8,9,23,28,30,31] found red areas in 16/141 patients (11.35%), this appearance being an unusual manifestation of the disease. Finally, only 2 of 24 (8.33%) [8,9] collect the presence of ulcerated areas, which were observed in only one of the 11 cases included in their series (9.09%), and none of the studies included in our scoping review refer to the fissured or smooth appearance of the lesions.

Our next research question addresses *the extent to which the gingiva or palate are required to be involved in order to classify a lesion as PVL*. In Cerero-Lapiedra’s criteria [11], the gingiva and palate involvement is considered as a major not mandatory criterion, Carrard et al. [12] classify the location as suggested but not mandatory, while neither of the two remaining proposals [10,13] consider this question as a diagnostic criterion; 10/24 papers [8,9,21,23,24,26,28,31,33,36] analyzed in this scoping review (41.67%) offer data on the location of the lesions in 145 patients. The oral mucosal site mostly affected in patients with PVL was buccal mucosa (63/145 patients; 43.45%) followed by gingiva (58/145 patients; 40%), tongue (47/145 patients; 32.41%), and palate (29/145 patients; 13.79%). The results indicate that the gingiva is a frequent location for PVL lesions, although it is not considered mandatory for the diagnosis of this OPMD, the tendency to affect the palate is much less marked. A proposal for diagnostic criteria should not consider gingival or palatal involvement as a determining criterion, although it should be of diagnostic support, especially with regard to gingival involvement.

Another research question is related to *what extent it is necessary to demonstrate the malignant transformation of a white lesion in order to consider it as a PVL*. All of the four diagnostic criteria for PVL proposed malignant transformation as a common fact in the PVL evolution, although not required for diagnosis [10,11,12,13]; 100% of studies included in our current paper (24/24) [4,6,7,8,9,10,13,21,22,23,24,25,26,27,28,29,30,31,32,33,34,35,36,37] also corroborate this proposition, i.e., no study indicates malignancy as a mandatory criterion for diagnosis. Papers published in this regard by the WHO Collaborating Center for Oral Cancer [5] describe PVL as an aggressive form of leukoplakia, among other reasons due to its tendency to become malignant and to develop multiple carcinomas. We have reported that perhaps the malignant rate of PVL, close to 50% of the cases [1,5], could be underestimated due to inconsistent criteria used for diagnosis; thus, in our previous paper [5] it was shown that studies carried out with high methodological quality reported significantly higher malignant transformation rates. Furthermore, an underestimation of the malignancy rate is expected as a consequence of the short follow-up periods of patients in the series published [5], what seems logical because this irreversible and persistent OPMD could, at least theoretically, become malignant at any moment along its evolution. Thus, one might wonder what the reported malignant transformation rate for PVL would be if all patients in all series had been followed for life. From what has been commented, it should be concluded that the high risk of oral cancer in PVL should be clearly indicated in a conceptual proposition, which will reinforce the message that must necessarily be translated, i.e., PVL must be carefully followed to achieve an early diagnosis of the more than probable phenomenon of malignancy.

We also considered to *what extent it is necessary for the PVL diagnosis its resistance to any form of treatment*, understanding as such the lesion recurrence/reappearance after treatment. Two of the four proposals for diagnostic criteria for PVL published consider resistance to treatment as a mandatory criterion [10,12], while Cerero-Lapiedra [11] propose it as a major not mandatory criterion. Only two papers (8.33%) [30,32] of those analyzed in this scoping review report data on treatment recurrence. Garcia-Pola et al. (2016) consider recurrence to treatment as a mandatory criterion, therefore, reporting 100% of recurrences (14 recurrences), while Garcia-Chias et al. (2014) consider it as a major but not mandatory criterion, reporting 25% of recurrences (10 recurrences in 40 treated PVLs). Recently, a systematic review and meta-analysis on PVL recurrence after treatment has been published [38]; the results are conclusive, 232/397 patients treated for PVL (pooled proportion, 67.2%), especially applying surgical excision with a cold scalpel or laser, recurred after treatment. It should be noted that the mean follow-up period reported in this meta-analysis was 6 years, which in practical terms could be considered sufficient to pick up any recurrence. In our opinion, the scientific evidence on this matter, although scarce, advises considering resistance to treatment as a support criterion to be included in a conceptual and diagnostic proposal.

Another of our research questions refers to *the extent to which the sex and age of patients are decisive for the diagnosis of PVL*. In relation to sex, among the published diagnostic criteria, only Cerero-Lapiedra [11] considers the female affectation as a non-required minor criterion; 23 of 24 studies [4,6,7,8,9,10,13,21,22,23,24,25,26,27,28,29,30,31,32,33,34,35,36] included in this scoping review (95.83%) reported data in relation to sex; 346/556 (62.23%) patients, for whom information is available, were women. The result clearly indicates that males and females are affected by the disease in a very similar way, and therefore, sex should not be considered as a mandatory diagnostic criterion. Regarding age, no author has proposed an age range as a diagnostic requirement. None of the published diagnostic criteria consider age as a determining factor for diagnosis; 23 out of 24 studies [4,6,7,8,9,10,13,21,22,23,24,25,26,27,28,29,30,31,32,33,34,35,36] included in our scoping review (95.83%) report data in relation to age, with the mean age of the patients in the published series ranging from 40.3 to 80.8 years, being 63.06 years the mean of means. From what has been published it can be concluded that PVL develops primarily in the second half of life, around 60 years of age, and therefore, in our opinion, this information should appear in a conceptual proposal, also behaving as a diagnostic support criterion.

We also considered to *what extent the absence of recognizable etiological factors, essentially tobacco use, is decisive in the diagnosis of PVL*. Only Cerero-Lapiedra et al. [11] consider the absence of tobacco consumption as a minor non-mandatory criterion, while none of the remaining proposals refer to this aspect; 20/24 studies [7,8,9,10,13,21,22,23,24,25,26,27,29,30,31,32,34,35,36,37] included in our analysis (83.33%) reported data in relation to tobacco consumption, thus, 178 out of a total of 463 patients with PVL (38.44%) were smokers, from which it can be concluded that smoking habit is frequent in PVL patients and its absence should not be considered as a diagnostic criterion.

Among our research questions, finally is *whether histopathological analysis of the lesions is necessary to make the diagnosis of PVL*. All the proposals for the PVL diagnosis [10,11,12,13] consider the histological study of the tissue as a mandatory criterion and 16 of the 24 studies (66.67%) [4,7,9,10,13,21,23,25,26,27,29,30,31,32,35,36] of the scoping review consider the histopathological analyses to be decisive to reach the diagnosis of a PVL. The histopathological data reported by these 16 studies that address this aspect are: hyperkeratosis—reported by 10 of 24 studies [13,21,23,24,25,26,30,31,32,33]—which is present in 101 of 143 patients included in their series (70.63%); atrophy—reported by one study [26]—that appeared in one patient out of a total of 15 (6.67%); acanthosis—reported by 2/24 studies [25,26]—which was found in 6 out of 18 patients (33.33%); lymphocytic infiltrate in the chorion, with a band arrangement—reported by 2 of 24 studies [23,31] in 3 of 10 patients (30%); verrucous hyperplasia—reported by 7 of 24 studies [8,9,13,23,31,32,36] in 38 of 87 patients included in their series (43.68%); epithelial dysplasia—reported by 10 of 24 studies [13,21,24,26,28,30,31,32,34,36] in 159 of 274 patients (58.03%); verrucous carcinoma, reported by 16 of 24 studies [9,10,13,21,22,23,24,25,26,27,30,31,32,35,36,37] in 81 of 384 patients (21.09%); papillary squamous carcinoma—reported by 3 of 24 studies [10,27,35] in 21 of 98 patients (21.43%); finally, squamous carcinoma—reported by 18 of 24 studies [4,8,9,10,13,21,22,23,24,25,26,27,30,31,32,35,36,37] in 139 of 472 patients (29.45%). As can be deduced from the results presented, the most common histological events are hyperkeratosis, epithelial dysplasia, verrucous hyperplasia, as well as the different forms of squamous carcinomas that are present; 21.09% of oral cancer in PVL are verrucous carcinomas, which are less aggressive, and so the prognosis of these patients could presumably be favorable. Future studies focused on this aspect should be conducted to offer results on the prognosis of carcinomas in these patients based on a higher quality of evidence. In our view, the inclusion of the histopathological study in the recommended procedures for the diagnosis of PVL is justified by the high frequency of epithelial dysplasia and malignant transformation of the lesions, although it is debatable that one or a set of histological features may be specific or highly suggestive of PVL, and it is generally recommended that histologic analyses could serve as diagnostic support in cases of compatible signs and to diagnose malignancy or evaluate the presence of dysplasia. Recently, an experts group with the support and approval of the American Academy of Oral and Maxillofacial Pathology and the North American Society of Head and Neck Pathologists has published a consensus guide to the histological features in PVL [39]. In this guide, four categories were considered to standardize and report the histopathological findings of PVL, namely: (1) “corrugated ortho (para) hyperkeratotic lesion, not reactive”; (2) “bulky hyperkeratotic epithelial proliferation, not reactive”; (3) “suspicious for” or “squamous cell carcinoma”; (4) “does not fit any above category”. Contrary to what we observed in our scoping review, in which epithelial dysplasia appeared in 58% of patients, the authors of this consensus guide report, without referring to any bibliographic citations, that the presence of epithelial dysplasia is a rare occurrence.

The potential limitations of the present scoping review are truly inherent to the primary-level studies investigated, being essentially the low sample sizes of most of the studies—which is fully justified given the low incidence of PVL—and the scarcity of data reported to answer some of the questions formulated (e.g., clinical appearance of PVL lesions). Future studies should make a comprehensive report of the characteristics of their patients, and the questions formulated by us could serve as a reporting checklist for the authors of future studies so as not to forget the approach to some essential aspects that should not be denied in the investigation of this OPMD. The main strength of our study is fundamentally due to its originality, this being the first scoping review that offers to address the diagnostic criteria of PVL in a holistic way and based on scientific evidence.

## 5. Conclusions

In conclusion, it is worth asking what a PVL is, or in other words, what clinicopathological aspects should be included in a concept proposal. In our view, and taking into account what was previously stated in the discussion, a *PVL is an oral potentially malignant disorder that presents in the form of multifocal white plaques, which have expanded throughout its evolution, persistent and resistant to treatment, which is diagnosed in people in the second half of life, although it probably begins in earlier stages, and which has a very high risk of developing oral cancer*. We must recognize that this concept only partially modifies, based on the evidence, the concept initially proposed by Hansen et al. [10], authors who demonstrated great insight in their initial analysis of this lesion. As can be deduced from our reflections in the discussion, some of the aspects of this conceptual proposition are based on the available evidence—i.e., the expansive behavior, the persistence of the lesions and their resistance to treatment—and require a long follow-up of the patients or the information extracted from a meticulous medical history. It should also be specified that from the histological analysis one should not wait to obtain specific facts of this entity, although it is essential to assess the presence of dysplasia and diagnose—early—cancer in suspicious areas. It is also important to think over when a clinician is authorized to make an accurate diagnosis of PVL on the first visit. The issue is transcendent since it implies assuming a very high risk of cancer and implementing specific management measures, including information to the patient. In our opinion, only exhaustive compliance with all the aspects included in our conceptual proposal, some of which must be extracted from the patient’s medical history, will allow to reach the maximum level of diagnostic certainty. Otherwise, it would be interesting to reflect on the level of certainty with which we made our diagnosis in those cases of less certain diagnosis. Thus, for example, an intermediate level of certainty could correspond to white lesions in patients in the second half of life, not too extensive, in which their recurrence after treatment and their expansive nature throughout their evolution have been demonstrated. A low level of certainty could correspond to white lesions that appear in the second half of life, essentially affecting a small limited area of the oral mucosa, with small incipient lesions appearing in other remote locations. It is evident that it is impossible to establish clinical facts that precisely define the diagnostic certainty levels of PVL since these are largely based on the experience of the clinician, although some facts could reinforce our level of certainty, i.e., the presence of warty areas and perhaps gingival involvement; however, we believe that it is necessary to reflect on the extent to which the diagnosis of PVL becomes important among the possibilities of presumed diagnosis of a white lesion, since as we commented, this implies establishing follow-up protocols and specific patient information. From what has been discussed so far, it is also deduced that it is very difficult, if not impossible, to establish a proposal of diagnostic criteria that encompasses all the possibilities with which a PVL can present itself from the beginning.

## Figures and Tables

**Table 1:** Diagnostic criteria published for proliferative verrucous leukoplakia (PVL).

CRITERIA/Conceptual Proposal	Definitions or Proposal for PVL Diagnostic Criteria
**Hansen et al. 1985** [10]	“…specific form of leukoplakia. It began as a simple hyperkeratosis but tended to extend and become multifocal over varying periods of time. The lesions were slow-growing, persistent, irreversible, and frequently developed erythematous components. Some areas later became exophytic and wart-like and transformed into lesions that were clinically and microscopically identical to verrucous carcinoma and squamous cell carcinoma. In addition, they were resistant to every kind of therapy.”
**Cerero-Lapiedra et al. 2010** [11]	Major Criteria (MC): A. A leukoplakia lesion with more than two different oral sites, which is most frequently found in the gingiva, alveolar processes and palate. B. The existence of a verrucous area. C. That the lesions have spread or engrossed during development of the disease. D. That there has been a recurrence in a previously treated area. E. Histopathologically, there can be from simple epithelial hyperkeratosis to verrucous hyperplasia, verrucous carcinoma or oral squamous cell carcinoma, whether in situ or infiltrating. Minor Criteria (mc): An oral leukoplakia lesion that occupies at least 3 cm when adding all the affected areas. That the patient be female. That the patient (male or female) be a non-smoker. A disease evolution higher than 5 years. Diagnosis of PVL: Three major criteria (being E among them) or Two major criteria (being E among them) + two minor criteria.
**Carrard et al. 2013** [12]	Leukoplakia showing the presence of verrucous or wart-like areas, involving more than two oral subsites. The following oral subsites are recognized: dorsum of the tongue (unilateral or bilateral), border of the tongue, cheek mucosa, alveolar mucosa or gingiva upper jaw, alveolar mucosa or gingiva lower jaw, hard and soft palate, floor of the mouth, upper lip and lower lip. When adding all involved sites, the minimum size should be at least three centimeters. A well-documented period of disease evolution of at least five years, being characterized by spreading and enlarging and the occurrence of one or more recurrences in a previously treated area. The availability of at least one biopsy in order to rule out the presence of a verrucous carcinoma or squamous cell carcinoma.
**Villa et al. 2018** [13]	White/keratotic lesions that may be smooth, fissured, verrucous, or erythematous with or without ulcer. Multifocal non-contiguous lesions or a single large lesion >4.0 cm involving one site or a single large lesion >3 cm involving contiguous sites. Lesions that progress/expand in size and/or develop multifocality over time. Histopathology that, if not overtly exhibiting dysplasia or carcinoma, shows hyperkeratosis, parakeratosis, atrophy, or acanthosis with minimal to no cytologic atypia (KUS), with or without a lymphocytic band, or verrucous hyperplasia; these features must not support a diagnosis of frictional or reactive keratoses
**Proposal by Gonzalez-Moles et al. 2021** [5] **derived from the evidence obtained in this scoping review**	PVL is an oral potentially malignant disorder that presents in the form of multifocal white plaques, which have expanded throughout its evolution, persistent and resistant to treatment, which is diagnosed in people in the second half of life, although it probably begins in earlier stages, and which has a very high risk of developing into oral cancer.

**Table 2:** Descriptive questions on proliferative verrucous leukoplakia diagnostic criteria, grouped in a matrix format and based on topic areas, to search for evidence-based results and potential evidence gaps.

Question 1	Is the clinical course of the disease (persistent or recurrent, periodicity of recurrences) determining in the PVL diagnosis?
Question 2	To what extent is the age of the lesion decisive for the diagnosis? Do the studies provide information on the age of the lesions? (follow-up time and/or months of evolution)
Question 3	What should be the clinical appearance of the lesions to make a diagnosis of proliferative verrucous leukoplakia? Were the clinical descriptions made by the authors of the lesions included in their incorporated cohorts?
Question 4	Is it necessary for the affection of gingiva and/or palate to make the diagnosis of proliferative verrucous leukoplakia? Were the anatomical affectations per patient reported from the included cohorts?
Question 5	Is it necessary to demonstrate malignant transformation to make the diagnosis of proliferative verrucous leukoplakia? Were the malignant transformation proportions reported in the included cohorts?
Question 6	Is resistance to treatment necessary to make the diagnosis of proliferative verrucous leukoplakia?
Question 7	To what extent is sex required to make the diagnosis of proliferative verrucous leukoplakia? Was the number of females and males reported in the included cohorts?
Question 8	To what extent is age required to make the diagnosis of proliferative verrucous leukoplakia? Was the age of patients reported in the included cohorts?
Question 9	To what extent is tobacco use or its absence necessary to make the diagnosis of proliferative verrucous leukoplakia? Was number of smokers and non-smokers reported in the included cohorts?
Question 10	Is the histological study necessary for the diagnosis of proliferative verrucous leukoplakia? What should be the histological substrate required to make the diagnosis of proliferative verrucous leukoplakia? Were the histological descriptions reported by the authors of the lesions analyzed incorporated in their cohorts?

**Table 3:** Study characteristics and reported data in primary-level studies (*n* = 24).

Author (Year)	Country	Diagnostic Criteria	Study Design (Recruitment Period)	Sample (*n*); Sex; Age Distribution (y) Mean ± SD (Range)	Anatomical Sites per Patients (*n*, Analysis Units = Patients, Not Lesions)	Malignant Transformation (*n*, %)	Malignant Transformation (High: >40%; Intermediate: 20–40%; Low: <20%)	Smoking	Clinical Diagnosis	Histological Diagnosis	Follow Up (Months)
McParland and Warnakulasuriya (2020) [34]	UK	Hansen et al. (1985) [10]	Retrospective cohort (2000–2016)	Sample size = 51 M = 25 (49.1%) F = 26 (50.9%) Mean age: 52.3 ± 8.65	NR	MT = 11 (21.57%) No MT = 40	Intermediate	Yes = 10 Former = 12 Never = 29 (56.86%)	NR	ED = 12	≤48
Li et al. (2021) [33]	USA	Own	Retrospective cohort (NR)	Sample size = 4 M = 2 (50%) F = 2 (50%) Mean age: 58 ± 21.74	Gingiva = 3 Bm = 4 Tongue = 4 Palate = 2 FOM = 1 Lip = 1 Other = 1	MT = 3 (75%) (SCC = 3 patients, 7 tumors) No MT = 4	High	NR	NR	HK = 4 Papillomatosis = 2 Corrugated = 2	Mean = 114
Favia et al. (2021) [37]	Italy	Hansen et al. (1985) [10]	Retrospective cohort (1989–2008)	Sample size = 75 Sex = NR Age = NR	NR	MT = 48 (64%) (VC = 33 patients, 57 tumors; SCC = 15 patients; 73 tumors) No MT = 27	High	Yes = 11 No = 64 (84.33%)	NR	NR	Mean = 62.45 Range = 18–240
Bagan et al. (2020) [4]	Spain	Villa et al. (2018) [13]	Retrospective cohort (1996–2018)	Sample size = 81 M = 29 (35.8%) F = 52 (64.2%) Mean age: 62.6 ± 12.3	NR	MT = 33 (40.74%) (SCC = 33 patients, 105 tumors) No MT = 48	High	NR	NR	NR	Mean = 65.61 ± 77.45 Range: 12–256.8
Koh and Kurago (2019) [36]	USA	Own	Retrospective cohort (NR)	Sample size = 10 M = 5 (50%) F = 5 (50%) Mean age: 60.7 ± 11.94	Gingiva = 6 Bm = 6 Tongue = 4 Palate = 2 FOM = 2 Lip = 1 Other = 0	MT = 5 (50%) (VC = 2, SCC = 2, SCC+VC = 1; 6 tumors) No MT = 5	High	Yes = 6 No = 2 (20%) Missing = 2	NR	VH = 10 ED = 8	Mean = 39.6 Range: 12–84
Upadhyaya et al. (2018) [35]	USA	Hansen et al. (1985) [10]	Retrospective cohort (1994–2016)	Sample size = 20 M = 6 (30%) F = 14 (70%) Mean age: 62.7 (range: 34–87)	NR	MT = 9 (45%) (VC = 6, PSCC = 1, SCC = 2) No MT = 11	High	Yes = 12 (60%) No = 5 (25%) NA = 3	NR	Grade 2 = 12 Grade 4 = 3 Grade 5 = 1	Mean = 91.8
Villa et al. (2018) [13]	USA and Brazil	Villa et al. (2018) [13]	Retrospective cohort (1996–2016)	Sample size = 42 M = 7 (16.7%) F = 35 (83.3%) Mean age: 67.23 ± 11.95	NR	MT = 30 (71.43%) (SCC = 25, VC = 5) No MT = 12	High	Yes = 5 Former = 12 Never = 24 (57.14%)	NR	HK = 22 ED = 17 VH = 5	Mean = 47.06 ± 47.33
Thomson et al. (2018) [28]	UK	NR	Retrospective cohort (1996–2014)	Sample size = 80 M = 41 (51.25%) F = 39 (48.75%) Mean age: 62.3 (range: 25–94)	Gingiva = 11 Bm = 15 Tongue = 19 Palate = 5 FOM = 16 Lip = 11 Other = 3	MT = 2 (2.5%) No MT = 78	Low	NR	White plaque = 80 Progressive = NR Multifocal = NR Slow growth = NR Erithematous = 2 Verrucous-like = NR Fissured = NR Ulcerated = NR	ED = 68	Mean = 87.6
Borgna et al. (2017) [27]	UK	Hansen et al. (1985) [10]	Retrospective cohort (1990–2015)	Sample size = 48 M = 24 (50%) F = 24 (50%) Mean age: 70 ± 13	NR	MT = 23 (47.92%) (VC = 10, SCC = 9, papillary SCC = 4) No MT = 25	High	Yes = 33 No = 15 (31.25%)	NR	Grade 2 = 2 Grade 3 = 14 Grade 4 = 14 Grade 5 = 9 Grade 6 = 5 Grade 8 = 1 Grade 9 = 3	Mean = 51.6 ± 44.4
Flores et al. (2016) [26]	Brazil.	Own	Retrospective cohort (NA)	Sample size = 15 M = 0 (0%) F = 15 (100%) Mean age: 68.13 ± 9.82	Gingiva = 9 Bm = 15 Tongue = 11 Palate = 5 FOM = 7 Lip = 3 Other = 1	MT = 4 (26.67%) (VC = 1, SCC = 5;6 tumors) No MT = 11	Intermediate	Yes = 0 (0%) No = 15 (100%)	NR	HK = 4 ED = 13 Acanthosis = 3 Atrophy = 1	Mean = 65.6 ± 63.15
García-Pola et al. (2016) [32]	Spain	Own	Prospective cohort (1984–2015)	Sample size = 14 M = 3 (21.4%) F = 11 (78.6%) Mean age: 56.4 (range: 35–69)	NR	MT = 4 (28.57%) (VC = 1, SCC = 3;12 tumors, 2 VCs, 10 SCCs) No MT = 10	Intermediate	Former = 3 No = 11 (78.57%)	NR	HK = 14 Papillomatosis = 10 VH = 9 ED = 1	Mean = 174
Ottavioli et al. (2016) [25]	France	Carrard et al. (2013) [12]	Retrospective cohort (NA)	Sample size = 3 M = 0 (0%) F = 3 (100%) Mean age: 80.7 ± 4.9	NR	MT = 2 (66.67%) (VC = 1, SCC = 1) No MT = 1	High	Yes = 0 (0%) No = 3 (100%)	NR	HK = 3 Papillomatosis = 3 Acanthosis = 1	Mean = 24 ± 12
Akrish et al. (2015) [29]	Israel	Own	Retrospective cohort (1990–2012)	Sample size = 11 M = 6 (55.5%) F = 5 (45.5%) Mean age: 64	NR	MT = 11 patients (38 SCCs) No MT = NR	NA	Yes = 1 No = 10 (90.91%)	NR	NR	>70
Thennavan et al. (2015) [8]	India	Own	Retrospective cohort (NR)	Sample size = 7 M = 1 (14.3%) F = 6 (85.7%) Mean age: 63.7 (range: 54–76)	Gingiva = 6 Bm = 7 Tongue = 2 Palate = 1 FOM = 0 Lip = 1 Other = 1	MT = 1 (14.29%) (SCC = 1; 1 tumor) No MT = 6	Low	Yes = 3 No = 4 (57.14%)	White plaque = 7 Progressive = 7 Multifocal = 7 Slow growth = NR Erithematous = 1 Verrucous-like = 2 Fissured = NR Ulcerated = 1	VH = 7 ED = 6	14
Owosho et al. (2015) [31]	USA	Cerero-Lapiedra et al. (2010) [11]	Retrospective cohort (2007–2013)	Sample size = 7 M = 4 (57.1%) F = 3 (42.9%): Mean age = 63.7 (range: 47–82)	Gingiva = 6 Bm = 6 Tongue = 2 Palate = 0 FOM = 0 Lip = 0 Other = 0	MT: 2 (28.57%) (1 VC, 1 SCC; 4 tumors, 1 VC, 2 SCCs, 1 hybrid VC/SCC) No MT: 5	Intermediate	Yes = 0 No = 7 (100%)	White plaque = 7 Progressive = NR Multifocal = 5 Slow growth = NR Erithematous = 2 Verrucous-like = 2 Fissured = NR Ulcerated = NR	HK = 6 Lymphocytic infiltrate = 2 VH = 2 ED = 7	Mean = 56.4
García-Chías et al. (2014) [30]	Spain	Cerero-Lapiedra et al. (2010) [11]	Retrospective cohort (1984–2011)	Sample size = 40 M = 15 (37.5%) F = 25 (62.5%) Mean age: 62.3	NR	MT = 7 (17.5%) (3 VC, 4 OSCC) No MT = 33	Low	Yes = 12 No = 28 (70%)	White plaque = 40 Progressive = 26 Multifocal = 38 Slow growth = NR Erithematous = 9 Verrucous-like = 14 Fissured = NR Ulcerated = NR	HK = 40 ED = 20	Mean = 44
Mehrotra et al. (2012) [6]	India	NR	Retrospective cohort (2007–2009)	Sample size = 3 M = 3 (100%) F = 0 (0%) Age: 40.3 ± 7.6	NR	MT = 0 (0%) No MT = 3	Low	NR	NR	NR	≤48
Morton et al. (2007) [23]	USA	NR	Retrospective cohort (NR)	Sample size = 3 M = 1 (33.33%) F = 2 (66.67%) Age: 80 ± 8.19	Gingiva = 2 Bm = 1 Tongue = 0 Palate = 1 FOM = 0 Lip = 1 Other = 1	MT = 3 (100%) (VC = 1, SCC = 2) No MT = 0	High	Yes = 1 No = 2 (66.67%)	White plaque = 3 Progressive = NR Multifocal = 1 Slow growth = NR Erithematous = 1 Verrucous-like = 1 Fissured = NR Ulcerated = NR	HK = 2 Lymphocytic infiltrate = 1 VH = 2	NR
Klanrit et al. (2007) [24]	UK	Own	Retrospective cohort (1990–1999)	Sample size = 6 M = 1 (16.67%) F = 5 (83.33%) Age: 65.83 ± 10.11	Gingiva = 6 Bm = 2 Tongue = 2 Palate = 3 FOM = 0 Lip = 1 Other = 0	MT = 6 patients (13 tumors VC = 2, cuniculatum = 3, SCC = 8) No MT = NR	NA	Yes = 1 Former = 1 No = 3 (50%) Missing = 1	NR	HK = 6 EP = 6 VH = 1	Mean = 116
Campisi et al. (2004) [22]	Italy	Own	Retrospective cohort (NR)	Sample size = 58 M = 22 (37.93%) F = 36 (62.07%) Age: 66.5 ± 12.92	NR	MT = 25 (43.10%) (VC = 3, SCC = 22) No MT = 33	High	Yes = 8 Former = 9 No = 41 (70.69%)	NR	NR	NR
Ghazali et al. (2003) [21]	Malaysia	Hansen et al. (1985) [10]	Retrospective cohort (NR)	Sample size = 9 M = 2 (22.22%) F = 7 (77.78%) Mean age: 61.67 ± 15.16	Gingiva = 6 Bm = 5 Tongue = 3 Palate = 1 FOM = 1 Lip = 1 Other = 0	MT = 7 (77.78%) (VC = 1, SCC = 3, VC+SCC = 3; 13 tumors, 5 VCs, 8SCCs) No MT = 2	High	Yes = 4 No = 5 (55.56%)	NR	VH = 3 ED = 7	Mean = 56.4
Zakrzewska et al. (1996) [7]	UK	Hansen et al. (1985) [10]	Retrospective cohort (NA)	Sample size = 10 M = 5 (50%) F = 5 (50%) Mean age (63.5, range: 42–81)	NR	MT = 10 (100%) No MT = 0	High	Yes = 7 No = 3 (30%)	NR	Grade 2 = 2 Grade 3 = 3 Grade 4 = 1 Grade 5 = 4	Mean = 79.2
Kahn et al. (1994) [9]	USA	NR	Retrospective cohort (1988–1990)	Sample (*n* = 4) M = 2 (50%) F = 2 (50%) Mean age = 68.75 (range: 51–75)	Gingiva = 3 Bm = 2 Tongue = 0 Palate = 0 FOM = 0 Lip = 0 Other = 1	MT = 3 (75%) No MT = 1	High	Yes = 2 No = 2 (50%)	White plaque = 4 Progressive = NR Multifocal = 3 Slow growth = NR Erithematous = 1 Verrucous-like = 3 Fissured = NR Ulcerated = 0	VH = 3 ED = 1	Mean = 48 Range = 24-60
Hansen et al. (1985) [10]	USA	Hansen et al. (1985) [10]	Retrospective cohort (1961–1983)	Sample (*n* = 30) M = 6 (20%) F = 24 (80%) Mean age = 49 (range: 27–74)	NR	MT = 27 (90%) (VC = 4, papillary = 18, SCC = 5) No MT = 3	High	Yes = 18 No = 12 (40%)	NR	Grade 3 = 1 Grade 4 = 2 Grade 5 = 1 Grade 6 = 3 Grade 7 = 6 Grade 8 = 12 Grade 9 = 2 Grade 10 = 3	Mean = 73.2

**Table 4:** Research questions considered in this scoping review, grouped in a matrix format, according to the essential thematic areas, and results related to the aforementioned questions raised in the evidence derived from the research on malignant transformation of PVL. The table shows how the four proposals recommended use of these items in their proposed diagnostic criteria. (yes—green/no—red answer).

Research Questions		Hansen et al. [10]	Cerero-Lapiedra et al. [11]	Carrard et al. [12]	Villa et al. [13]
**Q1**	- Is the clinical course of the disease (persistent or recurrent, periodicity of recurrences) determining in the PVL diagnosis?	Yes	No	No	No
**Q2**	- To what extent is the age of the lesion decisive for the PVL diagnosis?	No	Yes	Yes	No
**Q3**	- What should be the clinical appearance of the lesions to make a diagnosis of PVL?	Yes	Yes	Yes	Yes
	Clinical descriptions of the PVL lesions made by the authors: White plaque	Yes	Yes	Yes	Yes
	Multifocality	Yes	Yes	Yes	Yes
	Progressive/expansive nature	Yes	Yes	Yes	Yes
	Verrucous-like	No	No	Yes	No
	Erythematous areas	Yes	No	No	Yes
	Ulcerated areas	No	No	No	Yes
	Fissured appearance	No	No	No	Yes
	Smooth appearance	No	Yes	No	No
**Q4**	- Is it necessary the affectation of gingiva and/or palate to make the diagnosis of PVL?	No	Yes	Yes	No
	Intraoral sites affected by PVL lesions: Buccal Mucosa	No	No	No	No
	Gingiva	No	Yes	Yes	No
	Tongue	No	No	No	No
	Palate	No	Yes	Yes	No
**Q5**	- Is it necessary to demonstrate malignant transformation to make the diagnosis of PVL?	Yes	Yes	Yes	Yes
	- Malignant transformation reported	Yes	Yes	Yes	Yes
**Q6**	- Is resistance to treatment necessary to make the diagnosis of PVL?	Yes	No	Yes	No
**Q7**	- To what extent is sex required to make the diagnosis of PVL?	No	Yes	No	No
	Description per sex: Females	No	Yes	No	No
**Q8**	- To what extent is age required to make the diagnosis of PVL?	No	No	No	No
	Description of age reported	No	No	No	No
**Q9**	- To what extent is tobacco use or its absence necessary to make the diagnosis of PVL?	No	Yes	No	No
	Description for smoking habit: Non-smokers	No	Yes	No	No
**Q10**	- Is the histological study necessary for the diagnosis of PVL?	Yes	Yes	Yes	Yes
	Description of alterations in histology of PVL: Hyperkeratosis	Yes	Yes	No	Yes
	Atrophy	No	No	No	Yes
	Acanthosis	No	No	No	Yes
	Lymphocytic infiltrate in the lamina propria	No	No	No	Yes
	Verrucous hyperplasia	Yes	Yes	No	Yes
	Epithelial dysplasia	Yes	No	No	Yes
	Verrucous carcinoma	Yes	Yes	No	No
	Papillary carcinoma	Yes	No	No	No
	Squamous cell carcinoma	Yes	Yes	No	Yes

NA, not applicable; NR, not reported.

**Table 5:** Research questions considered in this scoping review, grouped in a matrix format, according to the essential thematic areas, and results related to the aforementioned questions raised in the evidence derived from the research on malignant transformation of PVL.

Research Questions		PVL Diagnostic Criteria Proposed Including Each Research Question *	Primary-Level Studies Included in This Scoping Review		
			Studies (*n*, %) Considering the Research Question among Their PVL Diagnostic Criteria	Patients (*n*) with Available Data **	Positive Cases (%) **
**Q1**	- Is the clinical course of the disease (persistent or recurrent, periodicity of recurrences) determining in the PVL diagnosis?	Hansen et al. (1985) [10]	6/24 (25%) (all persistent)	NR	NR
**Q2**	- To what extent is the age of the lesion decisive for the PVL diagnosis?	Cerero-Lapiedra et al. (2010) [11] Carrard et al. (2013) [12]	5/24 (20.83%)	NR	NR
**Q3**	- What should be the clinical appearance of the lesions to make a diagnosis of PVL?	All	NA	NA	NA
	Clinical descriptions of the PVL lesions made by the authors: White plaque	All	6/24 (25%)	141	141 (100%)
	Multifocality	All	6/24 (25%)	81	74 (91.36%)
	Progressive/expansive nature	All	3/24 (12.5%)	51	36 (70.59%)
	Verrucous-like	Carrard et al. (2013) [12]	5/24 (20.83%)	61	22 (36.07%)
	Erythematous areas	Hansen et al. (1985) [10] Villa et al. (2018) [13]	6/24 (25%)	141	16 (11.35%)
	Ulcerated areas	Villa et al. (2018) [13]	2/24 (8.33%)	11	1 (9.09%)
	Fissured appearance	Villa et al. (2018) [13]	0/24 (0%)	0	0 (0%)
	Smooth appearance	Cerero-Lapiedra et al. (2010) [11]	0/24 (0%)	0	0 (0%)
**Q4**	- Is it necessary the affectation of gingiva and/or palate to make the diagnosis of PVL?	Cerero-Lapiedra et al. (2010) [11] Carrard et al. (2013) [12]	1/24 (4.17%)	NA	NA
	Intraoral sites affected by PVL lesions: Buccal Mucosa	None	10/24 (41.67%)	145	63 (43.45%)
	Gingiva	Cerero-Lapiedra et al. (2010) [11] Carrard et al. (2013) [12]	10/24 (41.67%)	145	58 (40%)
	Tongue	None	10/24 (41.67%)	145	47 (32.41%)
	Palate	Cerero-Lapiedra et al. (2010) [11] Carrard et al. (2013) [12]	10/24 (41.67%)	145	29 (13.79%)
**Q5**	- Is it necessary to demonstrate malignant transformation to make the diagnosis of PVL?	All	0/24 (0%) ***	NA	NA
	- Malignant transformation reported	All	24/24 (100%)	631	266 (43.74%)
**Q6**	- Is resistance to treatment necessary to make the diagnosis of PVL?	Hansen et al. (1985) [10] Carrard et al. (2013) [12]	2/24 (8.33%)	54	24 (44.44%)
**Q7**	- To what extent is sex required to make the diagnosis of PVL?	Cerero-Lapiedra et al. (2010) [11]	2/24 (8.33%)	NA	NA
	Description per sex: Females	Cerero-Lapiedra et al. (2010) [11]	23/24 (95.83%)	556	363 (62.23%)
**Q8**	- To what extent is age required to make the diagnosis of PVL?	None	0/24 (0%)	NA	NA
	Description of age reported	None	23/24 (95.83%)	556 ****	Mean of means = 63.06y
**Q9**	- To what extent is tobacco use or its absence necessary to make the diagnosis of PVL?	Cerero-Lapiedra et al. (2010) [11]	2/24 (8.33%)	NA	NA
	Description for smoking habit:Non-smokers	Cerero-Lapiedra et al. (2010) [11]	20/24 (83.33%)	463	285 (61.56%)
**Q10**	- Is the histological study necessary for the diagnosis of PVL?	All	16/24 (66.67%)	NA	NA
	Description of alterations in histology of PVL: Hyperkeratosis	Hansen et al. (1985) [10] Cerero-Lapiedra et al. (2010) [11] Villa et al. (2018) [13]	10/24 (41.67%)	143	101 (70.63%)
	Atrophy	Villa et al. (2018) [13]	1/24 (4.17%)	15	1 (6.67%)
	Acanthosis	Villa et al. (2018) [13]	2/24 (8.33%)	18	6 (33.33%)
	Lymphocytic infiltrate in the chorion	Villa et al. (2018) [13]	2/24 (8.33%)	10	3 (30%)
	Verrucous hyperplasia	Hansen et al. (1985) [10] Cerero-Lapiedra et al. (2010) [11] Villa et al. (2018) [13]	7/24 (29.17%)	87	38 (43.68%)
	Epithelial dysplasia	Hansen et al. (1985) [10] Villa et al. (2018) [13]	10/24 (41.67%)	274	159 (58.03%)
	Verrucous carcinoma	Hansen et al. (1985) [10] Cerero-Lapiedra et al. (2010) [11]	16/24 (66.67%)	384	81 (21.09%)
	Papillary carcinoma	Hansen et al. (1985) [10]	3/24 (12.5%)	98	21 (21.43%)
	Squamous cell carcinoma	Hansen et al. (1985) [10] Cerero-Lapiedra et al. (2010) [11] Villa et al. (2018) [13]	18/24 (75%)	472	139 (29.45%)

NA, not applicable; NR, not reported., * This column reflects which diagnostic criteria for PVL proposed in the literature (Hansen et al. 1985 [10], Cerero-Lapiedra et al. 2010 [11], Carrard et al. 2013 [12] and Villa et al. 2018 [13]; described in Table 1) include the research question posed. ** Although a specific study may consider the research question posed as one of its diagnostic criteria, in some cases it does not offer individual data on the patients in its series. *** Although the 24 studies included in the scoping review offer the number of malignant cases in their series, none consider malignant transformation as a diagnostic criterion for the disease. **** Most of the studies that report data on age offer the mean of the series, with very few individual patient data on age.

## Data Availability

Data sharing not applicable to this article as no datasets were generated or analyzed during the current study.

## Supplementary Materials

The following are available online at https://www.mdpi.com/article/10.3390/cancers13153669/s1, File S1: protocol.
