# Supplementary material for: A Scoping Review on Gaps in the Diagnostic Criteria for Proliferative Verrucous Leukoplakia: A Conceptual Proposal and Diagnostic Evidence-Based Criteria
Source: Cancers (Basel). 2021 Jul 21;13(15):3669. doi: 10.3390/cancers13153669 (PMC8345058; doi:10.3390/cancers13153669)
Supplement: Supplementary file 1 [file cancers-13-03669-s001.zip › cancers-1291261-supplementary.pdf]

PROTOCOL

Anticipated or actual start date.

February, 2021

Anticipated completion date.

July, 2021

Stage of the scoping review at Feb 2021

Review stage

Preliminary searches: Completed

Piloting of the study selection process: Completed

Formal screening of search results against eligibility criteria: Started

Data extraction: Not strated

Data analysis: Not started

Funding sources/sponsors.

None

Conflicts of interest.

None

Review question.

Are diagnostic criteria for proliferative verrucous leukoplakia based on evidence? Do these criteria respond to the clinical and histopathological reality of proliferative verrucous leukoplakia?

Searches

Studies published in PubMed, Embase, Web of Science and Scopus (upper limit=February 2021). Thesaurus terms (e.g., MeSH or Emtree) will not be used due to a lack of specific terms for proliferative verrucous leukoplakia. Preliminary searches were conducted by combining the keywords “proliferative” AND “verrucous” AND “leukoplakia”. An additional screening will also be performed handsearching the reference lists of the retrieved included studies. All references will be managed using Mendeley v.1.19.4 (Elsevier. Amsterdam, The Netherlands); duplicate references will be eliminated using this software.

Condition or domain being studied.

Proliferative verrucous leukoplakia is considered as an oral potentially malignant disorder with a high malignant transformation proportion. Nevertheless, recent meta-analyses have shown a remarkable variability in terms of the incidence of oral cancer in patients with proliferative verrucous leukoplakia, mainly attributed the imprecision and discrepancies between the proposed diagnostic criteria for this disease. Based on this background, the evaluation of current knowledge and gaps of evidence of current criteria for proliferative verrucous leukoplakia diagnosis seems pertinent.

Type and method of review

Scoping review

Scoping review framework

Following the Condition, Context and Population CoCoPop framework -specifically designed from Joanna Briggs Institute (University of Adelaide, Australia)- the following elements and elegibility criteria were considered for inclusion: cohorts studies investigating the proportion of cases with malignant transformation (condition); in individuals with proliferative verrucous leukoplakia diagnosed by clinical and/or histopathological criteria (context); and their related characteristics (e.g., sex, age, tobacco use, anatomical sites affected and age of the lesions, clinical course, resistance to treatment, etc).

Types of study to be excluded:

Studies not investigating proliferative verrucous leukoplakia or not researching its malignant transformation proportion will be excluded; Studies focused on gingival

affections; Cross-sectional, clinical trials, case reports, reviews or meta-analyses, commentaries or personal opinions, hypotheses, protocols, letters, posters, meeting abstracts, and preclinical research (*in vitro* studies and/or animal experimentation); and/or overlapping populations.

Outcome(s)/endpoint(s):

The concept of proliferative verrucous leukoplakia and evidence-based diagnostic criteria will be explored through critical analysis and evidence synthesis in a scoping review.

Data extraction (selection and coding).

Data will be extracted data across future identified and selected articles, using Excel and Word (v.16/2018, Microsoft. Redmond, WA) data collection forms in a standardized way. Data expressed as medians, maximum-minimum values and/or interquartile ranges) will be computed and transformed into means±SDs using Luo *et al.* (2018) and Wan *et al.* (2014) methods. Data will be gathered on the first author, year of publication, country, publication language, sample size, follow up and recruitment periods, study design (i.e., prospective or retrospective cohorts), diagnostic criteria, location of proliferative verrucous leukoplakia (patient will be the analysis unit), age and sex, tobacco consumption.

Critical analysis and evidence synthesis

Descriptive questions (see below), grouped in a matrix format and based on topic areas, to search for evidence based-results and potential evidence gaps were designed, and will be were critically appraised for each individual study. Then, results and issues will be synthetized and discussed, to reach consensus for each question.

Evidence-based results will be obtained and potential evidence gaps will be identified where insufficient research evidence exists about a particular topic. Finally, recommendations and statements will be formulated.

Design of questions

The following descriptive questions on proliferative verrucous leukoplakia diagnostic criteria were grouped, based on topic areas, to search for evidence based-results and potential evidence gaps:

-Is the clinical course of the disease determining in the diagnosis?

-Does the author communicate if the clinical course is persistent or recurrent?

-If so, does it provide information on the periodicity of recurrences?

-To what extent is the age of the lesion decisive for the diagnosis?

- Does the study provide information on the age of the lesions? (follow-up time, months or years)

- What should be the clinical appearance of the lesion to make a diagnosis of proliferative verrucous leukoplakia?

To answer this question, the clinical descriptions made by the authors of the lesions that they include in their series will be incorporated.

- Is the histological study necessary for the diagnosis of proliferative verrucous leukoplakia?

- What should be the histological substrate required to make the diagnosis of proliferative verrucous leukoplakia?

- Is it necessary to demonstrate malignancy to make the diagnosis of proliferative verrucous leukoplakia?

Here in this section we will include, in addition to the answer to the question, the data that the authors include on the carcinomas that have developed.

- Is it necessary for the lesion to be resistant to treatment to make the diagnosis of proliferative verrucous leukoplakia?

- Is it necessary that the gingiva and / or palate be affected to make the diagnosis of proliferative verrucous leukoplakia?

- Is it necessary to demonstrate that the lesions are multifocal at some point in their evolution to make the diagnosis of proliferative verrucous leukoplakia?

- Is it necessary to show that the lesions have spread to make the diagnosis of proliferative verrucous leukoplakia?

- To what extent is sex necessary to make the diagnosis of proliferative verrucous leukoplakia?

- To what extent is age necessary to make the diagnosis of proliferative verrucous leukoplakia?

- To what extent is tobacco use or its absence necessary to make the diagnosis of proliferative verucous leukoplakia?
